# The Gut Microbiota as a Mediator Linking the MIND Diet to Alzheimer’s Disease

**DOI:** 10.3390/nu18091445

**Published:** 2026-04-30

**Authors:** Fatemeh Ramezani, Sina S. Herfeh, Emily Burke

**Affiliations:** 1 Department of Food Service and Clinical Nutrition, Cedars-Sinai Medical Center, Los Angeles, CA 95136, USA; 2 Department of Biomedical Engineering, Azad University, Rasht 4147654919, Guilan, Iran

**Keywords:** MIND diet, microbiota–gut–brain axis, Alzheimer’s disease

## Abstract

The Mediterranean-DASH Intervention for Neurodegenerative Delay (MIND) diet has emerged as a promising dietary pattern associated with reduced Alzheimer’s disease (AD) risk, supported by growing evidence that both diet and the gut microbiota are modifiable contributors to disease development and progression. Observational studies have linked higher MIND diet adherence to lower AD incidence and slower cognitive decline, with certain comparative analyses reporting stronger associations with cognitive outcomes than those observed for the parent Mediterranean or DASH diets. Developed specifically to support cognitive health, the MIND diet emphasizes leafy green vegetables, berries, and olive oil while restricting butter, cheese, fried foods, sweets, and red meat. While these features suggest a biologically plausible basis for neuroprotection, the underlying mechanisms remain incompletely defined. The microbiota–gut–brain axis offers a potential mechanistic framework, as diet is a major determinant of gut microbiota composition and microbiota-derived metabolites that may influence brain function and AD-related pathways. However, direct evidence characterizing MIND diet-specific effects on the gut microbiota remains limited, with most mechanistic insights derived from related dietary patterns or individual dietary components. Accordingly, this review synthesizes evidence from these related dietary patterns and key MIND components to propose a conceptual framework linking the MIND diet, the gut microbiota, and AD risk, while highlighting priorities for future research.

## 1. Introduction

The global burden of Alzheimer’s disease (AD) continues to escalate, placing an immense strain on healthcare systems and affected families [1]. Given the limited efficacy of pharmacological interventions in halting disease progression, attention has increasingly shifted toward preventive and modifiable lifestyle factors [2]. Greater adherence to multiple healthy lifestyle behaviors has been associated with a substantially lower risk of AD [3]. Among these factors, diet has received particular attention due to its central role in shaping metabolic and inflammatory pathways [4], including those mediated through the microbiota–gut–brain axis, which is an increasingly recognized pathway in AD pathogenesis [5].

Early ecological and multi-country studies suggested that higher intake of total fat and animal products may increase AD risk, whereas fish and cereal consumption may exert protective effects [6,7,8]. These findings prompted a shift from isolated nutrients toward overall dietary patterns, recognizing that cumulative dietary exposures better capture real-world eating behaviors [9]. Within this context, the Mediterranean-DASH Intervention for Neurodegenerative Delay (MIND) diet was devised to selectively integrate dietary components that may be associated with reduced AD risk and slower cognitive decline [10,11], with certain comparative analyses reporting stronger cognitive associations than its parent Mediterranean or DASH patterns [12,13,14]. Despite this epidemiological evidence [15], the mechanisms through which the MIND diet may confer neuroprotection remain incompletely understood [16,17].

One potential mechanistic pathway involves the gut–brain axis. Historically, the gut has been described as the “second brain” due to the autonomy of the enteric nervous system [18]. However, this concept extends beyond neural control alone. More recently, the trillions of microorganisms residing in the gastrointestinal tract, collectively termed the gut microbiota, have been recognized as central regulators of this bidirectional communication [19]. Through neural, immune, endocrine, and metabolic pathways, the gut microbiota influences neuroinflammation, neurotransmitter production, and blood–brain barrier (BBB) integrity, collectively forming what is known as the microbiota–gut–brain axis [19]. Given that AD is characterized by chronic neuroinflammation, barrier disruption, and dysregulated neuroimmune signaling, gut dysbiosis, defined as an imbalance in microbial composition or functional activity, has emerged as a potential contributor to AD pathogenesis [19,20,21]. Importantly, diet is a major determinant of gut microbiota composition [5], positioning the microbiota–gut–brain axis as a key interface linking dietary patterns to AD-related processes [19,20,22]. However, direct evidence characterizing the effects of the MIND diet on AD through microbiota-mediated mechanisms remains limited.

Accordingly, this review synthesizes evidence from parent dietary patterns and key MIND diet components to propose a conceptual framework linking this dietary pattern to gut microbial pathways relevant to AD and to highlight priorities for future research.

## 2. Methodology

This narrative review was conducted using a structured literature search of PubMed, Scopus, and Web of Science to identify studies relevant to the microbiota–gut–brain axis, dietary patterns, and AD pathogenesis, supplemented by manual screening of reference lists. Search terms included “gut microbiota”, “microbiome”, “microbiota–gut–brain axis”, “short-chain fatty acids”, “Alzheimer’s disease”, “neuroinflammation,” “amyloid”, “tau”, “Mediterranean diet”, “DASH diet”, “MIND diet”, and key MIND diet components such as “berries”, “leafy green vegetables”, “extra-virgin olive oil”, “nuts” and “fish”.

Eligible studies included observational cohorts, randomized and non-randomized dietary interventions, and mechanistic studies (animal and in vitro), as well as review articles and meta-analyses that provided insight into diet–microbiome–brain interactions. Given the limited number of studies directly evaluating the MIND diet in relation to the gut microbiome, evidence from parent dietary patterns and individual dietary components was integrated to inform mechanistic pathways. Findings were synthesized qualitatively to develop a conceptual framework linking the MIND diet, gut microbial pathways, and AD risk, and to identify key evidence gaps and priorities for future research.

## 3. The Microbiota–Gut–Brain Axis

**Figure 1:**
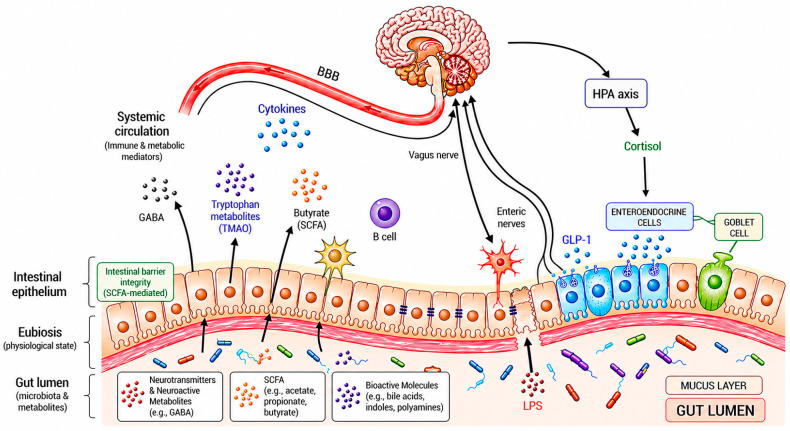
Overview of the microbiota–gut–brain axis.

The microbiota–gut–brain axis is a dynamic, bidirectional communication network in which gut microbial communities and their metabolites influence central nervous system (CNS) function while simultaneously receiving regulatory input from the brain [19] as illustrated in Figure 1. Within the context of diet-microbiota interactions, this axis represents a key mechanistic pathway linking dietary patterns such as the MIND diet to AD-related processes. Ascending (gut-to-brain) communication occurs through several interconnected pathways.

The microbiota-gut–brain axis is a bidirectional communication network linking the gut microbiota and the central nervous system through neural (vagus nerve), endocrine, immune, and metabolic pathways. In the gut-to-brain direction, microbial metabolites (e.g., short-chain fatty acids (SCFAs), trimethylamine N-oxide (TMAO)), cytokines, neurotransmitters (GABA), and enteroendocrine signals influence neuroinflammation, blood–brain barrier (BBB) integrity, and synaptic function. In the brain-to-gut direction, outputs from the hypothalamic–pituitary–adrenal (HPA) axis and the autonomic nervous system regulate intestinal permeability, motility, and microbial composition. Created by the authors in Microsoft PowerPoint with AI-assisted visual refinement.

Gut microbes contribute to neurochemical regulation by modulating the biosynthesis and turnover of key neurotransmitters, including γ-aminobutyric acid (GABA), serotonin, and dopamine, which affect neural signaling and brain function [23]. In addition to neurotransmitter modulation, microbial metabolism generates a diverse array of bioactive compounds, most notably short-chain fatty acids (SCFAs), primarily butyrate, acetate, and propionate, as well as secondary bile acids. These metabolites can interact with enteroendocrine and enterochromaffin cells, regulate host immune and inflammatory responses, and indirectly influence neural circuits [24,25]. Notably, certain SCFAs can cross or signal across the BBB and influence cerebral metabolism through multiple mechanisms, including epigenetic regulation, maintenance of BBB integrity, and modulation of mitochondrial function. They may also serve as supplementary cerebral energy substrates under conditions of reduced glucose availability—a state characterized by cerebral glucose hypometabolism and insulin resistance in AD [26].

The gut microbiota also shapes host immune responses by regulating the balance of pro- and anti-inflammatory cytokines, which can activate the hypothalamic–pituitary–adrenal (HPA) axis and influence CNS immune surveillance [19,24]. Central to this immune signaling are microorganism-associated molecular patterns, such as lipopolysaccharide (LPS), which contribute to immune maturation under physiological conditions but may promote systemic inflammation and neuronal stress when dysregulated [27,28].

Neural communication represents another key route of ascending signaling. The vagus nerve provides a rapid conduit through which microbial metabolites and enteroendocrine signals reach brain regions involved in mood regulation, stress responsiveness, and cognition, while also modulating neuroinflammatory pathways [19,29].

Complementing these ascending pathways, the descending (brain-to-gut) arm of the axis reflects the brain’s regulation of peripheral physiological processes that influence the microbial ecosystem. A critical pathway involves activation of the HPA axis, which triggers the release of stress hormones, such as cortisol. These hormones exert direct effects on the gut, altering motility, permeability, and secretion and thereby promoting changes in microbial composition and diversity [19,30]. The autonomic nervous system, through sympathetic and parasympathetic efferents, further modulates intestinal barrier integrity, immune activity, and enteric nervous system function, thereby influencing the gut microbial ecosystem [19,31].

Beyond these physiological mechanisms, the brain can also influence the gut microbiota indirectly through behavioral regulation. Mood states can shape behavioral patterns such as dietary adherence, food choices, and physical activity [32,33], all of which may influence microbial diversity and susceptibility to gut dysbiosis.

Given these interconnected, bidirectional signaling pathways that provide a mechanistic framework for disease-associated microbial signatures, the next question is whether AD is associated with a pattern of gut microbial alterations.

## 4. AD-Associated Gut Microbial Signatures

With AD now defined by biomarker-based classifications that enable diagnosis decades before symptom onset [34], attention has shifted toward identifying early peripheral and microbial signatures associated with these biological alterations. Amyloid-β (Aβ) accumulation begins approximately 18 years before cognitive decline, followed by tau pathology and neurodegeneration [35]. The AT(N) framework, encompassing Aβ, tau, and neurodegeneration, has been extended to an ATX(N) system incorporating inflammatory, metabolic, and blood-based markers that reflect broader systemic processes involved in disease progression [34,36]. Within this framework, Aβ pathology (A) is characterized by Aβ42, Aβ40, and the Aβ42/Aβ40 ratio; tau pathology (T) by phosphorylated tau isoforms, particularly p-tau181, p-tau217, and p-tau231; and neurodegeneration (N) by markers of neuronal injury, including total tau (t-tau) and neurofilament light chain [34]. Within this evolving biomarker paradigm, the microbiota–gut–brain axis has emerged as a complementary perspective, highlighting gut dysbiosis as a potential contributor to early disease processes [20,37]. Mechanistically, disruptions in gut microbiota composition may contribute to AD pathogenesis by promoting neuroinflammatory responses [38]. Metagenomic analyses have revealed that more than 10% of microbial species and gene families exhibit significant compositional changes detectable from the earliest preclinical stages of AD [39]. Notably, several studies report associations between gut microbial signatures and biomarkers of Aβ and tau pathology during preclinical stages, whereas correlations with markers of neurodegeneration appear less consistent, suggesting that microbiota alterations may precede substantial neuronal loss [37].

**Figure 2:**
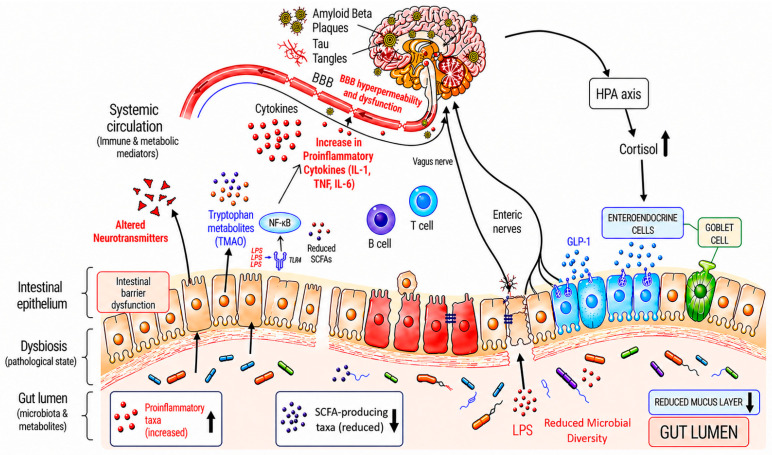
AD-associated gut dysbiosis and potential mechanisms in AD pathogenesis.

AD-associated gut dysbiosis is characterized by reduced microbial diversity, altered community composition, and perturbations in microbial metabolite profiles [38,40,41] across the disease spectrum, from mild cognitive impairment to dementia [42,43], and more broadly includes: (1) depletion of SCFA-producing bacteria; (2) enrichment of pro-inflammatory and LPS-producing bacteria; (3) reduced overall microbial diversity; and (4) perturbations in key microbial metabolic pathways [20,44] (Figure 2). Despite robust evidence of gut microbiota perturbations across the AD spectrum, no individual microbial taxa or metabolites have been definitively established as universal biomarkers of the disease, likely due to substantial heterogeneity across studies related to geography, diet, analytical methodologies, and population characteristics [42].

Gut dysbiosis in Alzheimer’s disease (AD) is characterized by a reduction in beneficial taxa (e.g., *Bifidobacterium*, *Faecalibacterium*), an expansion of pro-inflammatory species (e.g., *Escherichia/Shigella*), and decreased microbial diversity. These alterations contribute to increased intestinal permeability, facilitating the translocation of bacterial endotoxins such as lipopolysaccharide (LPS) and amyloidogenic proteins into systemic circulation. These gut-derived factors activate TLR4/NF-κB signaling pathways, promoting the release of pro-inflammatory cytokines and driving neuroinflammation. This cascade is associated with enhanced amyloid-β aggregation, tau hyperphosphorylation, and disruption of the blood–brain barrier, collectively contributing to neurodegeneration and cognitive decline. Arrows indicates direction of changes. Created by the authors in Microsoft PowerPoint with AI-assisted visual refinement.

Consistent with this pattern, AD-associated dysbiosis is marked by depletion of beneficial, SCFA-producing and fiber-fermenting taxa, particularly within the phylum Firmicutes, as well as reduced abundance of the genus *Bifidobacterium* (phylum Actinomycetota), alongside increased relative abundance of Bacteroidota and Proteobacteria, although geographic variability has been observed [42,45,46]. Key SCFA-producing bacteria include *Faecalibacterium prausnitzii*, *Roseburia*, *Eubacterium rectale*, select member of *Ruminococcus*, *Blautia*, and members of the Clostridiaceae and Lachnospiraceae families [44,46,47]. These organisms degrade complex polysaccharides and generate SCFAs either directly or through cross-feeding interactions [48,49].

Concurrently, AD-associated microbiota exhibit enrichment of pro-inflammatory, Gram-negative bacteria, including members of Proteobacteria, and genera such as, *Bacteroides*, *Escherichia*, and *Shigella*, which are positively correlated with peripheral inflammatory markers [38,47]. Notably, the abundance of the family Enterobacteriaceae progressively increases from healthy controls through mild cognitive impairment to AD, and may help distinguish individuals with AD from both preclinical stages and healthy individuals [50].

Mechanistic studies further suggest that specific microbial taxa may directly influence AD pathology. Members of the phylum Bacteroidota, particularly *Bacteroides fragilis* and its metabolites, have been shown to suppress microglial phagocytic function, impairing Aβ clearance and promoting plaque accumulation in mouse models [51]. Conversely, antibiotic depletion of Bacteroidota reduces amyloid load and activates microglial pathways related to phagocytosis and lysosomal degradation [51]. Oral pathogens, including *Porphyromonas gingivalis* and *Treponema denticola*, have also been reported to induce AD-like pathology, including Aβ plaques, hyperphosphorylated tau, neuroinflammation, and neuronal damage following chronic oral inoculation in mouse models [52,53].

These AD-associated microbial signatures extend beyond broad compositional imbalance and are associated with specific microbial taxa and metabolic pathways linked to AD neuropathology [19], thereby providing a foundation for examining potential gut microbiota-mediated mechanisms in AD pathogenesis.

## 5. Potential Gut Microbiota-Mediated Mechanisms in AD Pathogenesis

Emerging evidence suggests that the gut microbiota contributes to AD through multiple mechanisms within the gut–brain axis. Microbial metabolites influence neuroinflammation, BBB integrity, and neuronal signaling [20,54,55]. Key pathways linking gut dysbiosis to AD pathogenesis include SCFA signaling, microbial modulation of neurotransmitters, and immune activation via LPS signaling. Furthermore, alterations in host metabolic pathways involving secondary bile acids, trimethylamine-N-oxide (TMAO), and lipid metabolism may contribute (Figure 2).

### 5.1. SCFAs Signaling Pathway

Reduced abundance of SCFA-producing bacteria lowers SCFA levels—particularly butyrate, generated via microbial fermentation of dietary fiber in the colon [56], while polyphenols may further influence SCFA production through microbiota modulation [57]. Despite some inconsistencies [58], SCFAs exert neuroprotective effects through multiple mechanisms mediated primarily by histone deacetylase HDAC inhibition and activation of free fatty acid receptors. They maintain BBB integrity by upregulating tight junction proteins, and suppress neuroinflammation via inhibition of key signaling pathways, including the nuclear factor-kappa B (NF-κB) and mitogen-activated protein kinase (MAPK) pathways, collectively reducing pro-inflammatory cytokine expression and promoting anti-inflammatory microglial polarization [38,59,60]. They also influence cerebral energy metabolism and insulin sensitivity through gut-derived hormones, such as glucagon-like peptide-1 (GLP-1) [61]. Emerging evidence further suggests that SCFAs—particularly butyrate—may contribute to cerebral energy metabolism by modulating brain glucose utilization; preclinical studies indicate oxidative metabolism of butyrate in astrocytes supporting energy production and glutamine synthesis, as well as uptake and metabolism in AD models [26].

Preclinical evidence indicates that depletion of butyrate-producing bacteria precedes AD-related pathology, whereas butyrate reduces Aβ accumulation, neuroinflammation, tau pathology, and cognitive decline [62,63]. SCFA supplementation may also improve cognition independently of amyloid reduction [64]. Human studies similarly link reduced SCFA-producing taxa with increased amyloid burden and phosphorylated tau [65]. These findings support a mechanistic role for SCFAs in AD pathogenesis through barrier preservation, metabolic regulation, and modulation of neuroinflammatory and amyloidogenic processes.

### 5.2. LPS and Immune Activation

Circulating LPS levels are significantly elevated in individuals with AD compared to controls, with evidence reporting approximately threefold higher concentrations, although the magnitude varies across studies [66]. Derived from various gut bacterial taxa, LPS is a potent endotoxin that disrupts intestinal tight junction proteins and promotes systemic endotoxemia [67,68]. Upon entering the circulation, LPS activates innate immune responses primarily through Toll-like receptor 4 (TLR4), triggering downstream inflammatory signaling pathways including NF-κB and MAPK [69]. This chronic inflammatory milieu may accelerate core AD pathologies, including increased Aβ production and aggregation, impaired Aβ clearance, tau hyperphosphorylation and propagation, synaptic dysfunction, and progressive neurodegeneration [66]. Importantly, experimental models show that chronic peripheral LPS exposure can induce AD-like neuropathology even in wild-type mice with initially intact BBB structure, suggesting that sustained endotoxemia may initiate or potentiate neurodegenerative processes prior to overt BBB disruption [70].

### 5.3. Neurotransmitter Pathway

The gut microbiota plays a central role in regulating the synthesis of several neurotransmitters within the microbiota–gut–brain axis, with dysbiosis implicated in AD-related neurochemical disturbances [55]. Intestinal microbes synthesize and metabolize key neurotransmitters—including GABA, serotonin, dopamine, and glutamate—thereby influencing neural signaling through neural, immune, and metabolic pathways [23]. Disruptions in microbial composition may alter neurotransmitter availability and contribute to deficits observed in AD [71].

Specific gut taxa drive these processes. For instance, *Bacteroides* and *Lactobacillus* species are key microbial producers of GABA, and shifts in their abundance have been linked to AD-associated dysbiosis [72]. Specifically, *Bifidobacterium longum* 1714 enhances serotonin release through tryptophan metabolism modulation, while *Bacteroides finegoldii* increases the expression of tryptophan hydroxylase 1 (Tph1) in enterochromaffin cells [73,74]. Other genera, including *Coprococcus*, *Subdoligranulum*, and *Eggerthella*, have been linked to mood and cognitive performance [75]. Some microbial metabolites can enter systemic circulation and influence synaptic signaling, neuroinflammation, and oxidative stress, thereby contributing to AD pathogenesis [76].

### 5.4. Metabolic Pathway

*Trimethylamine-N-oxide (TMAO):* Dietary choline and carnitine, abundant in red meat and other animal-derived foods, are metabolized by intestinal microbiota—including members of the genera *Prevotella*, *Desulfovibrio*, and families Lachnospiraceae and Ruminococcaceae—to produce trimethylamine (TMA), which is subsequently oxidized in the liver by flavin-containing monooxygenase 3 (FMO3) to form TMAO, a metabolite associated with adverse neurocognitive outcomes [77]. Mechanistically, TMAO may promote protein aggregation via molecular crowding and activate NF-κB and the NOD-like receptor family pyrin domain-containing 3 (NLRP3) inflammasome, contributing to mitochondrial dysfunction, oxidative stress, and synaptic damage [78,79,80]. Elevated circulating TMAO levels have been reported in individuals with cognitive impairment and AD and correlate with biomarkers of neurodegeneration [81]. TMAO can also cross the BBB, further promoting neuroinflammation and cognitive decline [82]. Additionally, TMAO activates protein kinase RNA-like endoplasmic reticulum kinase (PERK) [83] and impairs β-cell function, reducing glucose-stimulated insulin secretion [84]. The resulting insulin resistance may exacerbate AD pathology by reducing Aβ clearance, promoting GSK-3-β-mediated tau hyperphosphorylation, disrupting cerebral glucose metabolism, and amplifying neuroinflammation [85,86].

*Secondary Bile Acids:* Secondary bile acids, produced through gut microbial conversion of primary bile acids, regulate neuroinflammation and blood–brain barrier integrity via farnesoid X receptor (FXR) and Takeda G protein-coupled receptor 5 (TGR5) signaling [87,88]. FXR is a nuclear receptor that regulates bile acid metabolism and suppresses inflammatory signaling, primarily by inhibiting NF-κB-mediated cytokine production, whereas TGR5 is a membrane G protein-coupled receptor that modulates immune responses through inhibition of the NLRP3 inflammasome [89]. They also influence brain function indirectly through gut-derived hormones such as GLP-1 [90]. In AD, bile acid profiles are altered, with reduced primary and increased secondary bile acids, particularly deoxycholic acid and its conjugates [91,92]. The deoxycholic acid-to-cholic acid ratio—a marker of microbial 7α-dehydroxylation—is strongly associated with cognitive decline and AD biomarkers, including Aβ, phosphorylated tau, brain atrophy, and cerebral hypometabolism [91,93].

*Kynurenine-tryptophan Pathway:* Tryptophan metabolism through the kynurenine pathway links immune activation to neurodegeneration and is partly regulated by the gut microbiota [94]. Microbial activity modulates tryptophan availability and inflammatory signaling, influencing the activity of indoleamine 2,3-dioxygenase (IDO) and tryptophan 2,3-dioxygenase (TDO) activity and increasing the kynurenine-to-tryptophan ratio, which is associated with neuroinflammation and cognitive decline in AD [95,96]. Downstream metabolites such as quinolinic acid and 3-hydroxykynurenine promote oxidative stress and excitotoxicity, whereas kynurenic acid exerts neuroprotective effects via modulation of glutamatergic signaling [95,97]. Microbiota-derived metabolites, particularly butyrate, may suppress IDO activation, while dysbiosis promotes inflammatory signaling and shifts kynurenine metabolism toward neurotoxic intermediates implicated in AD pathogenesis [98,99,100].

*Omega-3 Fatty Acid-Derived Microbial Metabolites:* Omega-3 polyunsaturated fatty acids (PUFAs), particularly docosahexaenoic acid (DHA) and eicosapentaenoic acid (EPA) from fatty fish, interact with the gut microbiota to generate bioactive lipid mediators with potential relevance to AD [101,102]. Gut microbes metabolize these dietary PUFAs into oxylipins and further modify them into diols, contributing to inflammatory and immune regulation [103]. These metabolites serve as precursors for specialized pro-resolving mediators (SPMs), including resolvins, protectins, and neuroprotectin D1, which promote the resolution of inflammation and may mitigate neuroinflammation in AD [104]. Beyond their role as precursors, DHA and EPA directly influence AD pathology by reducing Aβ production and aggregation, attenuating tau hyperphosphorylation, modulating microglial activation, and supporting neuronal membrane integrity and synaptic function [105].

*Oleic Acid and Microbiota-Derived Metabolites:* Oleic acid, a monounsaturated fatty acid abundant in olive oil, avocados, and nuts, suppresses Aβ fibril formation and attenuates neuroinflammation via inhibition of NF-κB signaling [106,107]. Intestinal microorganisms, including *Enterococcus faecalis*, *Bifidobacterium longum*, and *Lactobacillus acidophilus*, can metabolize dietary oleic acid via isomerization to produce trans-10-octadecenoic acid [108]. In addition, oleoylethanolamide (OEA), an oleic acid-derived lipid amide, enhances microglial Aβ clearance, suppresses inflammasome activation, and support a beneficial gut microbial profile and intestinal barrier integrity, linking oleic acid metabolism to neuroinflammatory regulation in AD [109,110].

*Sphingolipid and Ceramide Metabolism:* Ceramides are bioactive sphingolipids implicated in AD pathogenesis and are jointly regulated by diet and the gut microbiota. Saturated fat-derived palmitic acid promotes de novo ceramide synthesis, whereas prebiotic fibers (e.g., inulin) and omega-3 PUFAs reduce circulating and long-chain ceramides [111,112,113]. The gut microbiota further modulates systemic ceramide pools. Certain *Bacteroidetes* species encode serine palmitoyltransferase (SPT) and produce sphingolipids that increase hepatic ceramide levels [114], while reduced microbial diversity has been associated with elevated circulating ceramides and insulin resistance [115]. Through the liver–gut–brain axis, ceramides may contribute to neuroinflammation and neurodegeneration [116]. Mechanistically, ceramides promote Aβ production and aggregation, impair insulin signaling, and activate GSK-3β-mediated tau hyperphosphorylation [117].

*Homocysteine Metabolism:* The gut microbiota regulates homocysteine metabolism, a pathway linked to AD risk. Elevated plasma homocysteine is a modifiable risk factor, with each 5 μmol/L increase associated with ~15% higher AD risk [118]. Microbial communities influence homocysteine homeostasis through B-vitamin metabolism, particularly folate (B9), B12, and B6, which are essential for its degradation. Notably, 40–65% of gut bacteria possess B-vitamin biosynthetic pathways, with species such as *Lactobacillus reuteri* and *Bifidobacterium* contributing to de novo folate and cobalamin synthesis [119,120,121]. Disruptions in microbial composition may reduce vitamin availability and promote hyperhomocysteinemia [122,123]. Elevated homocysteine contributes to AD pathogenesis via Aβ aggregation, oxidative stress, vascular dysfunction, and neuronal ferroptosis [124].

Collectively, this evidence suggests that the gut microbiota may play a role in modulating AD-related molecular pathways and represents a potential target for dietary and therapeutic interventions. Within this context, dietary patterns such as the MIND diet have emerged as promising approaches for modulating the gut microbiota in ways that may influence AD risk and progression.

## 6. The MIND Diet: A Brain-Focused Mediterranean Dietary Pattern

Scientific investigations of dietary patterns in cardiometabolic and cognitive health have largely focused on the Mediterranean and DASH dietary models. Research on the Mediterranean diet originated within a cardiometabolic prevention framework, beginning with the Seven Countries Study in the 1960s, which demonstrated lower coronary heart disease mortality in Mediterranean populations [125]. The Mediterranean pattern emphasizes fruits, vegetables, whole grains, legumes, nuts, olive oil as the principal fat source, moderate fish and fermented dairy intake, limited red meat, and wine in moderation [125]. Similarly, the DASH (Dietary Approaches to Stop Hypertension) diet emerged from a multicenter randomized trial in the early 1990s to evaluate dietary effects on blood pressure [125]. The DASH diet emphasizes fruits, vegetables, low-fat dairy, whole grains, poultry, fish, and nuts, while limiting saturated fat, red meat, and sweets [126]. A distinguishing feature is its nutrient profile, with potassium, magnesium, and calcium levels approaching the 75th percentile of U.S. consumption through nutrient-dense foods [125].

The MIND diet, developed by Morris and colleagues in 2015 [10,11], represents a brain-directed modification of these two dietary models. This dietary pattern integrates key elements of both Mediterranean and DASH dietary patterns while selectively prioritizing foods with neuroprotective potential. It emphasizes extra-virgin olive oil and fish from the Mediterranean diet, restricts saturated fat and refined sugar from the DASH diet, and distinctively prioritizes green leafy vegetables and berries, foods associated with favorable cognitive outcomes and, in some studies, reduced Aβ deposition [15,16,125]. The MIND score comprises 15 components: 10 brain-healthy food groups (green leafy vegetables, other vegetables, nuts, berries, beans, whole grains, fish, poultry, olive oil, wine) and 5 unhealthy groups (red meat, butter/margarine, full-fat cheese, pastries/sweets, fried/fast food) [15]. Each component contributes up to 1 point (range 0–15), with adherence typically categorized as low (<7.0), moderate (7.0–8.9), or high (9.0–15.0) [16].

The Mediterranean diet remains the most extensively studied dietary pattern in cognitive aging research [9], with meta-analyses demonstrating inverse associations with mild cognitive impairment, dementia, and AD [127,128,129]. Postmortem evidence indicates that both MIND and Mediterranean patterns are associated with lower AD pathology and Aβ load [15]. Systematic reviews further show that higher MIND diet adherence correlates with better cognitive performance, slower decline, and reduced dementia incidence [16,130,131]. Some comparative analyses suggest a modestly stronger association for the MIND diet than for the Mediterranean or DASH diets alone [12,14,131,132], supporting the hypothesis that its neuroprotective components may act synergistically.

The neuroprotective potential of the MIND diet is thought to arise from the combined biological effects of its plant-rich components. These foods provide bioactive compounds—antioxidants, polyphenols, and unsaturated fatty acids—that may synergistically support vascular health, reduce oxidative stress, and attenuate inflammation [16,17,133]. The protective effects of the MIND diet extend beyond overall dementia incidence to specific cognitive domains, particularly episodic memory and global cognition [134,135]. These benefits persist after adjustment for AD neuropathology, suggesting cognitive resilience through pathways partially independent of amyloid and tau accumulation [134]. Proposed pathways include anti-inflammatory and antioxidant effects of key phytochemicals [125,136,137] as well as microbiota-mediated mechanisms [5,20,138]. Given the emerging role of the gut microbiota in these processes, evidence from the parent dietary patterns of the MIND diet provides important insight into potential neuroprotective microbial signatures.

## 7. Neuroprotective Microbial Signatures Associated with the Parent Diets of the MIND Diet

Given the limited direct evidence on microbiota-mediated neuroprotective effects of the MIND diet, current mechanistic insights are largely inferred from studies of individual MIND components and related dietary patterns. The Mediterranean diet, in particular, provides an important reference framework for understanding diet–microbiota interactions relevant to neurodegeneration [5], although these dietary patterns are not interchangeable.

Mediterranean dietary patterns are reported to be associated with greater microbial diversity and enrichment of SCFA-producing taxa. Several taxa that are enriched with Mediterranean diet adherence but reduced in AD, including *Lachnoclostridium*, *Parabacteroides*, *Roseburia*, *Ruminococcus*, *Slackia*, unclassified *Blautia*, and *Parabacteroides distasonis* are commonly implicated in fiber fermentation and SCFA production. In contrast, genera such as *Bilophila*, *Shigella/Escherichia*, and *Streptococcus* tend to be reduced with Mediterranean diet adherence yet elevated in AD [136]. Functional evidence supports the metabolic relevance of these compositional shifts. Postprandial increases in butyric acid following Mediterranean diet intervention have been inversely associated with insulin levels and positively correlated with insulin sensitivity [139]. In a randomized controlled trial, Mediterranean diet adherence was associated with increased fecal butyrate and reductions in plasma LPS-binding protein and fecal zonulin, suggesting improved intestinal barrier integrity and reduced LPS translocation [140]. A large multicenter trial in older adults found that a one-year Mediterranean diet intervention beneficially modified gut microbiota composition, increased beneficial microbial taxa, and was associated with reduced inflammation and improvements in cognitive-related outcomes [141]. Consistent with these findings, higher Mediterranean diet adherence has been linked to slower global cognitive decline, mediated in part by a distinct microbial signature enriched in SCFA-producing taxa and reduced pro-inflammatory taxa [137]. These microbiota shifts are also associated with lower circulating markers of metabolic endotoxemia, including LPS, as well as reduced levels of the microbiota-derived metabolite TMAO [142,143,144,145].

Comparable microbiota-related effects have been observed with the DASH dietary pattern. Higher DASH adherence has been associated with enrichment of fiber-fermenting taxa such as *[Eubacterium] eligens* and *[Eubacterium] xylanophilum*, suggesting enhanced fermentation of plant-derived polysaccharides and improved metabolic profiles [146]. Randomized trials further report reductions in circulating TMAO, LPS, and LPS-binding protein, indicating improved gut barrier integrity and reduced endotoxemia with DASH-based interventions [147,148].

These compositional and functional microbiota shifts provide a biological rationale for the MIND diet, which is derived from the DASH and Mediterranean dietary patterns and shares similar components, thereby potentially mitigating AD-related dysbiosis. The following section focuses on neuroprotective microbial signatures associated with key MIND diet components.

## 8. Neuroprotective Microbial Signatures Associated with Key MIND Diet Components

MIND diet individual components may provide a practical mechanistic basis for understanding how this dietary pattern may modulate gut microbiota and influence AD-related pathways. These individual components—including berries, leafy green vegetables, fish, olive oil, and nuts—are examined for their capacity to modulate gut microbial composition, enhance SCFA production, and attenuate gut-derived neuroinflammation.

### 8.1. Berry-Mediated Microbiota Modulation Relevant to AD

Berries are a characteristic component of the MIND diet and are rich sources of polyphenols. Berry polyphenols have been extensively investigated for their effects on gut microbiota composition and function, exerting prebiotic-like effects that may counteract AD-associated dysbiosis through enrichment of beneficial taxa and increased SCFA production [149,150]. Polyphenols are poorly absorbed in the small intestine, with most reaching the colon, where resident microbiota catabolize them into bioactive metabolites with enhanced bioavailability [151]. Accordingly, this bidirectional interaction, in which polyphenols shape microbial composition while microbes transform polyphenols into bioactive compounds, suggests that many of their health effects are mediated through microbial biotransformation [152]. Consistent with this mechanism, berry consumption increases the abundance of *Bifidobacterium*, *Lactobacillus*, and *Akkermansia muciniphila*, taxa commonly depleted in AD [149,153]. Beyond these compositional changes, berry polyphenols also modulate microbial metabolic outputs with relevance to neuroprotection. Anthocyanin-rich berry extracts alter tryptophan metabolism, increasing production of the neuroprotective metabolite kynurenic acid [154]. A meta-analysis further demonstrated that anthocyanin-rich diets significantly reduce the Firmicutes/Bacteroidetes ratio and enhance SCFA production [155]. In addition, colonic fermentation of berry polyphenols lowers luminal pH, enriches SCFA-producing taxa, and suppresses potentially harmful bacteria [156,157]. Anthocyanin-rich fruits also reduce pro-inflammatory cytokines, increase tight junction protein expression, and promote microbial shifts that may mitigate endotoxemia and neuroinflammation characteristic of AD [153]. These findings highlight berries as dietary modulators of the gut microbiota that may contribute to neuroprotection through the microbiota–gut–brain axis.

### 8.2. Green Leafy Vegetables-Mediated Microbiota Modulation Relevant to AD

Green leafy vegetables (GLVs), a characteristic component of the MIND diet, are rich in bioactive compounds, including nitrate, folate, vitamin K (phylloquinone), lutein, kaempferol, and dietary fiber, which may collectively modulate gut microbiota composition and function in ways relevant to AD pathogenesis. Dietary nitrate, highly concentrated in GLVs, undergoes a unique enterosalivary cycle dependent on oral and gut microbiota [158,159]. Nitrate-reducing bacteria in the oral cavity (e.g., *Veillonella*, *Actinomyces*, *Rothia*, and *Neisseria*) convert nitrate to nitrite, which is subsequently reduced to nitric oxide (NO) in the stomach and peripheral tissues [159,160]. This pathway is critical for NO homeostasis, with implications for cerebrovascular health and BBB integrity [161,162,163]. Thus, nitrate serves as a respiratory substrate for intestinal bacteria, where nitrate availability shapes microbial ecology while the microbiota regulate nitrate bioavailability, creating a bidirectional interaction that links GLV intake to microbial ecology and brain health [158].

Beyond nitrate metabolism, GLV-derived fiber exhibits distinct prebiotic effects. In murine models, kale increases microbial diversity, reduces the Firmicutes/Bacteroidetes ratio, and enriches Coriobacteriales and *Bacteroides thetaiotaomicron*, alongside enhanced glycan and metabolic pathways [164]. Spinach similarly increases diversity and alters composition, linked to linoleate and butanoate metabolism [165]. In human studies, vegetable intake—particularly GLVs—correlates with greater beta diversity and enrichment of fiber-degrading, SCFA-producing genera [166].

GLVs are the primary dietary source of folate, which interacts bidirectionally with the gut microbiota. Many gut bacteria synthesize folate de novo or from intermediates [121]. Supplementation increases *Lactobacillus*, *Bifidobacterium*, and *Pediococcus* and modulates SCFA profiles [167]. As these taxa are often depleted in AD, folate-rich GLVs may help restore beneficial microbial communities [20,168]. Importantly, folate functions as a key methyl donor in the remethylation of homocysteine to methionine; thus, adequate folate status—derived from dietary intake or microbial biosynthesis—is essential for preventing hyperhomocysteinemia, a recognized risk factor for AD [169].

GLVs contain flavonoids such as kaempferol that modulate gut microbial ecology. In murine models, kaempferol counteracts dysbiosis, enhances barrier integrity, suppresses TLR4-NF-κB signaling, and increases SCFA-producing bacteria, while also modulating bile acid pathways and enriching anti-inflammatory taxa [170,171]. These microbiota-mediated effects are relevant to AD, given links between dysbiosis, barrier dysfunction, and neuroinflammation [20,54].

Carotenoids in GLVs, particularly lutein, may modulate microbiota–gut–brain interactions. In vitro studies show that lutein increases SCFA production, promotes beneficial taxa, and inhibits pathogenic species [172]. It may also influence bacterial colonization via modulation of immunoglobulin A [173]. Epidemiologic data further link higher lutein intake to slower cognitive decline [174].

GLVs also contain thylakoids, chloroplast membrane structures with microbiota-modulating effects. A 3-month human intervention showed that thylakoid supplementation increased total bacterial abundance and *Lactobacillus*, while modulating satiety hormones [175]. These effects may support brain health via gut-mediated pathways, as *Lactobacillus* enhances hippocampal brain-derived neurotrophic factor (BDNF) and reduces neuroinflammation, and satiety hormones promote synaptic plasticity [176,177]. These data show that higher GLV intake may be associated with slower cognitive decline and better memory.

### 8.3. Extra-Virgin Olive Oil-Mediated Microbiota Modulation Relevant to AD

Extra-virgin olive oil (EVOO), the primary fat in the Mediterranean and MIND diets, contains monounsaturated fatty acids and phenolics (e.g., hydroxytyrosol, oleuropein, oleocanthal) that modulate gut microbiota and AD-related pathways [178,179]. EVOO exhibits prebiotic-like effects, increasing *Lactobacillus* and *Bifidobacterium* and enhancing SCFA production in vitro. Human studies further show increased Bacteroidota and reduced IL-1β following 12 weeks of daily EVOO consumption [180,181]. Microbial metabolism of EVOO phenolics generates bioactive compounds, including hydroxytyrosol, which suppresses neuroinflammation via TLR4-NF-κB and MAPK pathways and improves cognitive outcomes while attenuating Aβ pathology in preclinical models [179,182,183]. Epidemiologic and clinical evidence supports these cognitive benefits: higher EVOO intake is associated with improved cognition and microbial diversity, with specific taxa (e.g., *Adlercreutzia*) implicated as mediators. Furthermore, Mediterranean diet interventions supplemented with EVOO have been shown to prevent cognitive decline, and general olive oil intake is associated with a lower risk of neurodegenerative mortality [184,185,186].

Among EVOO phenolics, oleocanthal has been extensively studied for its effects on AD pathology. It enhances Aβ clearance across the BBB via upregulation of transport protein pathways [187]. Additionally, oleocanthal restores BBB function by inhibiting the NLRP3 inflammasome and activating autophagy [188]. Oleocanthal also preserves synaptic integrity by preventing Aβ-induced synaptic protein loss and neuroinflammation [189]. Preclinical evidence confirms that oleocanthal reduces Aβ burden and improves both metabolic and behavioral outcomes [190]. These findings position EVOO as a multifaceted neuroprotective agent that integrates gut microbiota modulation, anti-inflammatory signaling, and direct effects on AD pathology, supporting its role as a key dietary component for cognitive preservation in aging populations.

### 8.4. Nuts-Mediated Microbiota Modulation Relevant to AD

Nuts (e.g., walnuts, almonds, pistachios, cashews) are rich in fiber, polyphenols, and unsaturated fatty acids that exert prebiotic-like effects on gut microbiota relevant to AD [191,192]. Evidence indicates modest but consistent microbiota modulation, with walnuts showing the strongest effects on β-diversity [193,194]. Walnut intake increases butyrate-producing taxa (e.g., *Faecalibacterium*, *Roseburia*) and reduces pro-inflammatory bile acids [195]. Walnuts are particularly rich in ellagitannins (e.g., glansreginin A, pedunculagin, and casuarictin), which are metabolized by the gut microbiota into urolithins—bioactive compounds with high bioavailability and demonstrated neuroprotective effects [196]. Urolithins exert neuroprotective actions by upregulating BDNF signaling and protecting neurons against oxidative stress [193], as well as by attenuating LPS-induced neuroinflammation through inhibition of NF-κB and MAPK signaling pathways and reducing pro-inflammatory cytokines [197]. Urolithins further improve cognitive function in aging models by reducing Aβ accumulation and restoring synaptic function [198]. Notably, urolithins and their phase II conjugates cross the blood–brain barrier, preserve its integrity [194].

Other nuts show similar effects: almonds increase members of the Ruminococcaceae family and butyrate, pistachios more strongly enrich butyrate-producing bacteria, and cashew fiber exhibits high butyrogenic capacity [199,200,201,202].

Epidemiologic and clinical evidence links nut intake to cognitive outcomes and microbial diversity. A six-year study associated moderate nut consumption with slower cognitive decline and greater diversity, including enrichment of Lachnospiraceae UCG-004 linked to preserved cognition [203]. A randomized trial showed improved cognitive performance with mixed nuts alongside enrichment of a Lachnospiraceae variant [204]. Similarly, midlife nut intake was associated with lower late-life cognitive impairment risk, partly mediated by unsaturated fatty acids [205]. These findings suggest that nut consumption may beneficially modulate gut microbial composition and metabolite production, providing a plausible microbiota-mediated pathway linking nut-rich dietary patterns to reduced neuroinflammation and improved cognitive health.

### 8.5. Fish-Mediated Microbiota Modulation Relevant to AD

Fish provide bioactive compounds—including omega-3 PUFAs (EPA, DHA), selenium, vitamin D, and astaxanthin—that modulate gut microbiota, barrier integrity, and inflammation in pathways relevant to AD [206]. Higher fish intake is associated with reduced dementia and AD risk, with dose–response benefits [207].

Preclinical and in vitro evidence shows that omega-3 PUFAs counteract dysbiosis by enriching SCFA-producing taxa (e.g., *Lactobacillus*, Lachnospiraceae, *Bifidobacterium*, *Roseburia*, *Akkermansia*) and reducing LPS-producing bacteria, while increasing SCFAs and neuroactive metabolites [208]. In vitro fermentation studies confirm that omega-3 substrates increase *Akkermansia* spp., *Bifidobacterium* spp., and *Roseburia* spp. along with butyrate production and neuroactive metabolites (GABA, tyrosine, and phenylalanine) [209]. In transgenic mice, elevated tissue omega-3 levels reduce endotoxemia and systemic inflammation, mediated in part by increased intestinal alkaline phosphatase activity, which lowers LPS production and gut permeability [210]. Consistent with these mechanistic findings, interventional human data show that EPA/DHA supplementation increases SCFA-producing bacteria, including *Bifidobacterium*, *Roseburia*, and *Lactobacillus*, with reversible effects independent of administration route [206]. Observational studies further support these associations. In middle-aged and elderly women, serum DHA and total omega-3 levels were significantly correlated with higher gut microbial alpha diversity, with the strongest associations observed for operational taxonomic units from the Lachnospiraceae family, a butyrate-producing family commonly depleted in AD [211]. These associations were independent of dietary fiber intake, suggesting potential direct effects of omega-3 fatty acids on microbiota composition [211].

Omega-3 PUFAs also enhance gut barrier integrity via increased tight junction protein expression, and reduce TLR4-mediated inflammation, while exerting direct neuroprotective effects, including improved Aβ clearance, reduced tau pathology, and resolution of neuroinflammation [212,213,214].

Fish are an important source of selenium, which interacts with the gut microbiota, increasing diversity, enriching beneficial taxa such as *Akkermansia*, *Lactobacillus*, Lachnospiraceae, and Ruminococcaceae, and reducing Proteobacteria and pro-inflammatory cytokines, with associated cognitive benefits in aging models [215].

Fatty fish are among the richest dietary sources of vitamin D, a secosteroid hormone that modulates gut microbiota composition and barrier function. Evidence from systematic reviews and interventions shows that supplementation increases microbial diversity and enriches anti-inflammatory taxa (*Bifidobacterium*, *Lactobacillus*, and *Akkermansia*)—commonly depleted in AD [216,217]. Vitamin D also maintains intestinal barrier integrity via vitamin D receptor (VDR)-mediated signaling, which reinforces tight junction protein expression, ensures appropriate antimicrobial peptide levels, regulates antimicrobial peptide production, and modulates mucosal immune responses [218].

Astaxanthin, a lipophilic carotenoid concentrated in pink-fleshed fish, exhibits microbiota-modulating and barrier-enhancing effects in preclinical models. Supplementation increases *Akkermansia muciniphila* abundance, elevates plasma GLP-1, while reducing pro-inflammatory cytokines and NLRP3 inflammasome activation and upregulates tight junction proteins [219,220,221]. Although most evidence remains preclinical, these microbiota-modulating effects, combined with astaxanthin’s antioxidant properties, suggest a microbiota-mediated pathway linking fish consumption to neuroprotection. These mechanisms may explain why whole fish consumption shows more consistent cognitive benefits than isolated omega-3 supplementation, likely due to the broader nutrient matrix engaging complementary neuroprotective pathways [208,222,223].

These findings suggest that key components of the MIND diet may promote convergent changes in gut microbiota. Accordingly, the MIND diet may be associated with a microbial profile characterized by enrichment of SCFA-producing taxa, increased microbial diversity, and reduced abundance of pro-inflammatory and endotoxin-producing bacteria. These features are supported by the microbiota-modulating effects of its constituent components, as well as its overlap with Mediterranean and DASH dietary patterns. However, whether the MIND diet produces a microbial signature beyond that of its parent diets remains to be established.

## 9. Neuroprotective Microbial Signatures Associated with the MIND Diet

Although evidence remains limited, recent studies have begun to characterize gut microbial signatures associated with MIND diet adherence, as summarized in Table 1. Among the limited human studies linking the MIND diet to gut microbiota and cognition, the TwinsUK cohort study provides notable evidence. In this investigation of 509 female twins (mean age 51.9 ± 12.5 years; range 18–79), higher adherence to the MIND diet (mean score 7.6 ± 1.49; range 3–11.5) was associated with greater relative abundance of Ruminococcaceae UCG-010, a SCFA-producing taxon. Importantly, the abundance of this taxon was associated with less global cognitive decline over a 10-year follow-up period, suggesting a potential microbiota-mediated pathway linking diet quality to cognitive trajectories [223]. The co-twin design strengthened causal inference by controlling for genetic and early-life environmental confounders; however, microbiota data were cross-sectional, AD biomarkers were not assessed, and the all-female cohort with moderate MIND diet adherence limits generalizability to men and populations with higher dietary adherence.

Complementary cross-sectional evidence further characterizes the MIND diet microbial signature. In a study of 118 adults (aged 25–45 years) comparing MIND, Mediterranean, and DASH dietary patterns, beta diversity differed significantly across diets, indicating that overall microbial community structure varied according to dietary adherence [146]. MIND diet scores, together with relative abundance of *Eubacterium eligens* and fecal isobutyrate concentrations, were the best predictors of circulating triglyceride levels. Given that elevated midlife triglycerides are associated with poorer cognitive outcomes and increased AD pathology [224], these findings are consistent with a microbiota–metabolite pathway linking MIND diet adherence to long-term brain health [146]. *Eubacterium eligens*, a pectin-degrading Clostridia species, promotes anti-inflammatory production [225]. Notably, isobutyrate is a branched-chain fatty acid derived from amino acid fermentation rather than fiber fermentation, and its metabolic significance in this context requires further investigation, as elevated fecal isobutyrate has been associated with unfavorable lipid profiles in other studies [226]. Additional associations with Oscillospirales UCG-010—linked to lean metabolic phenotypes and resistant starch metabolism—and uncultured Rhodospirillales taxa suggest secondary ecological adaptations consistent with sustained, plant-forward, polyphenol-rich dietary exposure.

Beyond microbial composition, MIND-like dietary patterns may modulate neurotoxicmetabolic pathways. In a cross-sectional study of 88 older adults (aged 60–75 years) at risk of cognitive decline, MIND-NL diet adherence did not independently predict neuroinflammation or cognitive performance. However, MIND-NL adherence significantly moderated the relationship between systemic inflammation and neuroinflammatory metabolites, as well as between systemic inflammation and cognitive functioning. Detrimental associations between systemic inflammation and both neuroinflammation and cognition were observed only in individuals with lower MIND-NL adherence. Similarly, MIND-NL adherence moderated the relationship between intestinal barrier permeability neuroinflammation. Within participants with lower adherence, systemic inflammation mediated the relationship between intestinal barrier permeability and neuroinflammation [227]. In an exploratory analysis of 82 older adults at risk of cognitive decline from the HELI multidomain lifestyle intervention study, changes in adherence to the MIND-NL dietary pattern—a Dutch adaptation of the MIND diet that accounts for differences in food availability, portion sizes, and dietary habits between the United States and the Netherlands—were inversely associated with changes in the kynurenine-to-tryptophan ratio and the kynurenine-to-large neutral amino acids (LNAA) ratio over 26 weeks, suggesting that improved MIND-NL adherence may contribute to decreased activation of the kynurenine pathway [228]. In addition, MIND-NL adherence was associated with lower levels of the neurotoxic metabolite quinolinic acid, although this association was significant only in the unadjusted model and was attenuated after adjustment for lifestyle factors [228]. These protective effects likely reflect the combined anti-inflammatory, antioxidant, and microbiota-modulating effects of polyphenol-rich foods (berries, fruits, vegetables, and extra-virgin olive oil), omega-3 polyunsaturated fatty acids from fish, dietary fiber that promotes SCFA production, and B-vitamin-containing foods (leafy greens, whole grains, legumes), which may attenuate inflammation-driven IDO activity and shift tryptophan metabolism away from neurotoxic kynurenine pathway intermediates [229,230].

The available evidence, although preliminary, supports the biological potential of a microbiota-mediated pathway linking MIND diet adherence to cognitive preservation. However, studies that directly examine the triadic relationship among MIND diet adherence, gut microbiota composition, and AD-specific biomarkers are limited.

**Table 1:** Summary of studies examining associations between MIND diet adherence, gut microbiome signatures, and metabolic or cognitive outcomes relevant to AD.

Study/ Population	Design	Key Microbiome Findings	Metabolic/Clinical Associations	Strengths	Limitations
TwinsUK cohort (*n* = 509 female twins; *n* = 275 with 10-year cognitive follow-up) [223]	Observational (cross-sectional) microbiome at follow-up; 10-year longitudinal cognitive assessment	↑ Relative abundance of Ruminococcaceae UCG-010 (SCFA-producing taxon) with higher MIND diet adherence	Ruminococcaceae UCG-010 abundance associated with less decline in global cognition over 10 years; Each 1-point increase in MIND score associated with fewer errors in paired-associates learning	Co-twin design controls for shared genetics and early-life environmental factors; Monozygotic discordant twin analysis isolates dietary effects from genetic confounding; 10-year cognitive follow-up	Single time point; no AD biomarkers; all-female cohort limits generalizability; microbiome subsample analysis
Adults (*n* = 118; age 25–45 years) [146]	Cross-sectional; comparative analysis of MIND, Mediterranean, DASH, and HEI-2020 dietary patterns with 16S amplicon sequencing and targeted fecal metabolomics (volatile fatty acids, bile acids)	Distinct β-diversity across MIND, Mediterranean, DASH, and HEI-2020 patterns; ↑ Eubacterium eligens and SCFA-related taxa	MIND score + *Eubacterium* eligens abundance + fecal isobutyrate concentration best predicted triglyceride levels (R^2^ = 0.24)	Comparative dietary pattern analysis; integration of microbiome + metabolite data	Small sample; no cognitive outcomes; cross-sectional design, young adult population may not generalize to older at-risk populations
MIND-NL tryptophan metabolism study (*n* = 82; older adults at risk of cognitive decline [228]	Observational (baseline and 26-week follow-up within multidomain lifestyle intervention); MIND-NL adherence via FFQ; fasted plasma tryptophan metabolites measured at both time points	No direct microbiome profiling	MIND-NL adherence associated with lower levels of quinolinic acid in unadjusted model; changes in MIND-NL adherence were inversely associated with changes in kynurenine: tryptophan ratio and kynurenine: large neutral amino acids ratio, indicating ↓ activation of the neurotoxic kynurenine pathway; suggests MIND diet may shift tryptophan metabolism away from neurotoxic kynurenine pathway toward neuroprotective pathways	Longitudinal change-change analysis; links dietary pattern to specific neurotoxic metabolic pathway relevant to AD pathogenesis; mechanistic specificity (kynurenine pathway)	No direct microbiome profiling; small sample; embedded within multidomain intervention (cannot isolate diet effect); no cognitive outcome assessment
MIND-NL dietary pattern study (*n* = 88; age 60–75 years; at risk of cognitive decline) [227]	Observational cross-sectional; MIND-NL diet adherence assessed via FFQ; systemic inflammation, intestinal barrier permeability, and neuroinflammatory metabolites	No direct microbiome profiling;	Indirect microbiome-related effects: MIND diet adherence moderated the relationship between intestinal barrier permeability and neuroinflammation; within low-adherence participants, systemic inflammation mediated the intestinal permeability–neuroinflammation pathway	First human study linking MIND diet to gut–brain axis via intestinal permeability–systemic inflammation–neuroinflammation pathway; mediation analysis of gut permeability pathway	No direct microbiome profiling; small sample; cross-sectional design

## 10. Evidence Gap and Future Directions

**Figure 3:**
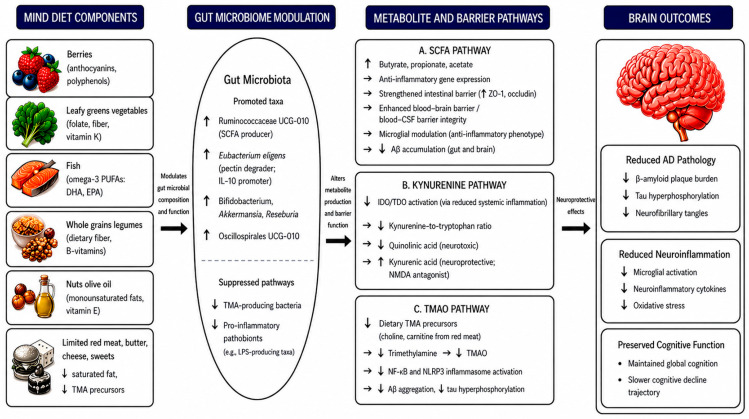
MIND Diet–Microbiota–AD Conceptual Model.

Intervention trials examining the MIND diet as a whole, rather than extrapolating from its parent diets or individual components, are essential to determine whether this dietary pattern produces a distinct microbial signature that mediates cognitive protection and mitigates AD pathogenesis (Figure 3). However, translating these findings into clinical practice requires addressing key methodological challenges.

Current observational approaches rely on food-frequency questionnaires, which introduce recall bias [231], while variable scoring systems complicate cross-study comparisons [232]. Future research should prioritize objective dietary assessment methods, including repeated digital dietary monitoring, nutritional metabolomics, and validated intake biomarkers, to mitigate bias inherent in self-reported data [185]. While observational data consistently suggest a protective association [135], the largest Randomized Controlled Trial (RCT) to date found no significant benefit over a calorie-restricted control at 3 years [17]. Notably, both the intervention and control groups received mild caloric restrictions and achieved comparable weight loss (~5 kg), which may have independently improved cognition across both arms, thereby masking diet-specific effects. Interestingly, a secondary analysis found that the MIND diet was associated with significantly higher cognitive scores among participants with BMI ≥ 35 kg/m^2^, suggesting potential benefits in this subgroup [233]. The overweight/obese study population (mean BMI ~34), COVID-19-related disruptions to trial operations, and practice effects from repeated cognitive testing represent additional factors that may have limited the trial’s ability to detect between-group differences. Future trials require mechanistic designs with biomarker endpoints, neuroimaging, longer durations, and “usual-diet” control groups. Furthermore, serial microbiota sampling and shotgun metagenomic sequencing will be critical to move beyond taxonomic profiling toward the functional characterization of microbial metabolic pathways [234]. Targeting at-risk midlife populations is equally vital, as this represents a window where AD pathology is emerging but still modifiable [16]. The role of sex and hormonal status remains underexplored, although the estrogen–gut microbiota–brain axis suggests that sex-specific stratification may optimize dietary interventions [235]. Multi-domain approaches are also essential. The US POINTER trial showed that combining the MIND diet with physical exercise and cognitive engagement improves cognition compared with self-guided strategies [236,237]. These findings support positioning high MIND diet adherence within a broader “muscle–gut–brain axis” framework. Additionally, lifestyle factors such as sleep and chronic stress, which influence the gut microbiota, are often unmeasured and may confound findings [238].

Ultimately, confirming the triadic relationship between MIND diet adherence, gut microbial ecology, and validated AD biomarkers represents the gold standard for future investigations [239]. Notably, blood-based biomarkers have demonstrated high accuracy in detecting AD pathology and can identify pathological changes up to two decades before symptom onset [234]. By adopting systems biology frameworks that integrate dietary patterns, metagenomics, hormonal profiles, and AD biomarkers, researchers can determine whether the gut microbiota causally mediates cognitive preservation—thereby clarifying the role of MIND diet within nutritional strategies for cognitive health and enabling personalized, microbiota-targeted interventions against AD.

The MIND diet, characterized by a high intake of plant-based foods and neuroprotective nutrients, may favorably modulate gut microbial composition by promoting beneficial taxa and their associated metabolites, including short-chain fatty acids (SCFAs), bile acid derivatives, and tryptophan metabolites, while reducing pro-inflammatory metabolites such as trimethylamine N-oxide (TMAO) and lipopolysaccharide (LPS). These compositional and functional shifts may enhance intestinal and blood–brain barrier integrity and attenuate systemic and neuroinflammation, amyloid-β accumulation, and tau pathology, thereby supporting brain health and potentially reducing the risk of Alzheimer’s disease (AD). However, no single study has yet established the complete causal pathway from MIND diet adherence through microbiome modulation to reduced AD pathology in humans. Created by the authors in Microsoft PowerPoint with AI-assisted visual refinement.

## 11. Conclusions

This review underscores the biological plausibility of the MIND diet as a potential modulator of the microbiota–gut–brain axis. By integrating the neuroprotective elements of the Mediterranean and DASH dietary patterns, the MIND diet may promote a distinct microbial environment characterized by enrichment of SCFA-producing taxa and a concomitant reduction in pro-inflammatory bacterial populations. These microbial shifts may contribute functional changes in gut-derived metabolites with potential neurobiological implications. Key dietary components of the MIND pattern may support intestinal barrier function, modulate systemic inflammatory responses, and influence metabolic pathways relevant to neuroprotection. However, to transition from observed associations to clinical utility, the field must overcome several methodological hurdles. Systems-level integration of nutritional, microbial, and biomarker data will be necessary to clarify causality, cementing the MIND diet’s role in preventive neurology and advancing microbiota-based AD therapeutics.

## Data Availability

No new data were created or analyzed in this study.

## Abbreviations

The following abbreviations are used in this manuscript:

AD	Alzheimer’s disease
MIND	Mediterranean–DASH Intervention for Neurodegenerative Delay
DASH	Dietary Approaches to Stop Hypertension
BBB	Blood–brain barrier
CNS	Central nervous system
GABA	Gamma-aminobutyric acid
SCFA(s)	Short-chain fatty acid(s)
HPA	Hypothalamic–pituitary–adrenal (axis)
LPS	Lipopolysaccharide
Aβ	Amyloid-beta
TMAO	Trimethylamine N-oxide
TLR4	Toll-like receptor 4
HDAC	Histone Deacetylase
MAPK	Mitogen-activated protein kinase
NF-κB	Nuclear factor kappa B
TLR-4	Toll-like receptor 4
TMA	Trimethylamine
FMO3	Flavin-containing monooxygenase 3
NLRP3	NOD-like receptor family pyrin domain-containing 3 (inflammasome)
PERK	Protein kinase RNA-like endoplasmic reticulum kinase
GSK-3-β	Glycogen synthase kinase 3 beta
FXR	Farnesoid X receptor
TGR5	Takeda G protein-coupled receptor 5
GLP-1	Glucagon-like peptide-1
IDO	Indoleamine 2,3-dioxygenase
TDO	Tryptophan 2,3-dioxygenase
EPA	Eicosapentaenoic acid
DHA	Docosahexaenoic acid
SPM	Specialized pro-resolving mediators
OEA	Oleoylethanolamide
SPT	Serine palmitoyltransferase
NO	Nitric oxide
GLV	Green leafy vegetables
EVOO	Extra-virgin olive oil
BDNF	Brain-derived neurotrophic factor
VDR	Vitamin D receptor
